# A systematic review of the microbiome of *Hyalomma* Koch, 1844 ticks using next-generation sequencing of the *16S ribosomal RNA* gene

**DOI:** 10.14202/vetworld.2025.1090-1100

**Published:** 2025-05-08

**Authors:** Mohamad Taha Al Masri, Mohammad Ali Al-Deeb

**Affiliations:** Department of Biology, United Arab Emirates University, Al-Ain, United Arab Emirates

**Keywords:** *16S ribosomal RNA* gene sequencing, *Hyalomma*, microbiome, Middle East and North Africa region, ticks

## Abstract

**Background and Aim:**

Ticks are critical vectors of pathogens affecting humans and livestock globally. The microbiome of ticks, comprising diverse bacterial communities, plays a crucial role in tick biology and vector competence. *Hyalomma* ticks are prominent in the Middle East and North Africa (MENA) region and are known carriers of significant pathogens. This study aimed to systematically evaluate existing literature regarding the microbiome composition of *Hyalomma* ticks in the MENA region, identify predominant bacterial genera, and highlight knowledge gaps.

**Materials and Methods:**

A systematic literature search was conducted using four databases: ScienceDirect, PubMed, Google Scholar, and Scopus. The search covered studies published between 2014 and 2024 employing *16S ribosomal RNA* gene sequencing to analyze microbiomes of *Hyalomma* ticks within the MENA region. Studies not fulfilling these criteria were excluded through independent assessment by two authors.

**Results:**

Out of 1,220 screened articles, seven studies met inclusion criteria, involving five *Hyalomma* species: *Hyalomma dromedarii*, *Hyalomma anatolicum*, *Hyalomma excavatum*, *Hyalomma marginatum*, and *Hyalomma scupense*. Most studies (57.14%) focused on *H. dromedarii*, primarily collected from camels. The geographical distribution of studies included the United Arab Emirates (42.86%), Saudi Arabia (28.57%), Iran (14.29%), and Tunisia (14.29%). Common bacterial genera identified across multiple studies included *Acinetobacter*, *Bacillus*, *Flavobacterium*, *Francisella*, *Rickettsia*, *Staphylococcus*, *Pseudomonas*, and *Corynebacterium*. However, substantial gaps were noted, particularly concerning variations related to tick lifecycle stages, host interactions, temporal dynamics, and extensive geographic coverage within the MENA region.

**Conclusion:**

This systematic review underscores the presence of key bacterial genera within *Hyalomma* ticks across the MENA region, revealing their potential roles in tick biology and pathogen transmission. Major research gaps identified include limited geographical scope, insufficient exploration of microbiome variation across tick life stages, host-specific interactions, and the environmental factors influencing microbial communities. Addressing these gaps through comprehensive, longitudinal, and multi-regional studies is imperative for improving public health strategies and developing targeted tick-control methods.

## INTRODUCTION

Ticks are obligate hematophagous arthropods widely distributed across the globe [1]. Taxonomically, ticks are categorized under the arachnid subclass Acari, within the superorder Parasitiformes and the order Ixodida, comprising three recognized families: Ixodidae (hard ticks), Argasidae (soft ticks), and Nuttalliellidae [2]. Ticks possess two primary body segments: the gnathosoma (also termed capitulum) and the idiosoma (body) [1]. Adult ticks have eight legs, while their larvae and nymphal stages have six legs [3]. These arthropods are significant vectors of diseases affecting humans, ranking second only to mosquitoes in their epidemiological importance [3]. A range of tick-borne pathogens has been identified, including protozoal pathogens such as *Theileria* and *Babesia*; bacterial pathogens such as *Rickettsia*, *Anaplasma*, and *Ehrlichia*; and viral pathogens including Nairobi Sheep Disease, Crimean-Congo Hemorrhagic Fever (CCHF), and flaviviruses [4, 5]. Moreover, ticks inflict considerable economic damage through the reduction of milk and meat yields, increased morbidity and mortality, and diminished quality of livestock hides and skins [6].

Considering these impacts, the study of ticks is crucial. The tick microbiome significantly influences tick survivability, fitness, developmental processes, immune responses, pathogenicity, and nutritional adaptation [7]. For instance, *Francisella* contributes to maintaining homeostasis [8] and synthesizing Vitamin B [9], while *Bacillus* and *Pseudomonas* mitigate pathogenicity [10]. In addition, *Rickettsia* performs provisional nutritional functions [11]. Thus, investigating the microbiome of ticks could offer valuable insights into managing their adverse effects. Next-generation sequencing of the *16S ribosomal RNA* (*16S rRNA*) gene is a primary technique in microbiome research [12–17], favored due to its presence in all bacteria, genetic stability, and adequate sequence length for precise identification at the genus level [13, 16]. This sequencing approach has effectively characterized the microbiome of *Hyalomma* ticks globally. Sequencing the V3-V4 hypervariable region of the *16S rRNA* gene has identified predominant bacteria, such as *Candidatus* *midichloria* and *Francisella*-like endosymbionts, in *Hyalomma anatolicum* from cattle in Pakistan [18], and *Francisella*-like endosymbionts in *Hyalomma* *lusitanicum* from roe deer in Spain [19]. Similarly, studies in Turkey employing this method have successfully described the microbiomes of three *Hyalomma* species, detecting dominance of *Francisella* and *Borrelia* in *Hyalomma aegyptium* from tortoises; *Escherichia*, *Curvibacter*, *Flavobacterium*, *Francisella*, *Paenibacillus*, and *Rickettsia* in *Hyalomma excavatum*; and *Escherichia*, *Curvibacter*, *Flavobacterium*, and *Rickettsia* in *Hyalomma marginatum*, with both latter species sampled from humans [20].

The genus *Hyalomma* Koch 1884 occurs primarily in the Palearctic, Oriental, and Afrotropical regions. Knowledge about this genus remains limited due to inaccuracies in contemporary identification keys. Nonetheless, considering *Hyalomma* species’ role as disease vectors [21], additional research is imperative. Detailed investigation of the microbiome of *Hyalomma* ticks can enhance understanding of their biology and endosymbiotic interactions, forming the basis for improved tick management strategies [22].

Despite the significant role that *Hyalomma* ticks play in transmitting critical tick-borne pathogens, the microbiome of these ticks remains understudied, particularly within the Middle East and North Africa (MENA) region. Existing microbiome research has predominantly focused on tick species in other regions, while only limited information is available for *Hyalomma* species native to MENA countries. The paucity of comprehensive studies addressing variations in micro-bial composition related to different tick species, life stages, host species, geographic locations, and temporal factors has restricted our understanding of tick-microbiome-pathogen dynamics. This knowledge deficit hinders the development of targeted management and control strategies for tick-borne diseases, which are of notable veterinary and public health concern in the region.

Therefore, this systematic review aims to comprehensively analyze and synthesize available literature on the microbiome composition of *Hyalomma* ticks within the MENA region using next-generation sequencing of the *16S rRNA* gene. Specifically, this review intends to identify predominant bacterial genera consistently reported across studies, elucidate their potential roles in tick biology and vector competence, and highlight critical gaps in the current research. By consolidating existing data and recognizing areas requiring further investigation, this review seeks to provide a robust foundation for future microbiome research, aiming to enhance understanding and inform effective tick management and disease prevention strategies in the region.

## MATERIALS AND METHODS

### Ethical approval

Ethical approval was not necessary, as this study was based solely on a systematic literature review. This systematic review was conducted and reported in accordance with the Preferred Reporting Items for Systematic reviews and Meta-Analyses 2020 guidelines for systematic reviews [23].

### Study period and location

Articles were retrieved in March 2024 at the United Arab Emirates (UAE) University, located in Al-Ain City, UAE.

### PICO framework

This systematic review was structured according to the PICO framework to ensure a focused and rigorous approach to the research question. The Population (P) included *Hyalomma* tick species across the MENA region, which are known vectors of several tick-borne diseases and of growing public and veterinary health concern. The Intervention (I) involved the application of *16S rRNA* gene sequencing, a widely accepted next-generation sequencing technique used to characterize the bacterial microbiome of ticks. The Comparison (C) was established across different *Hyalomma* species, host animals (e.g., camels, cattle, sheep, goats), and geographical locations within the MENA region to assess variation in microbiome composition. The Outcome (O) focused on identifying the predominant bacterial genera present in *Hyalomma* ticks, evaluating their ecological and pathogenic relevance, and detecting gaps in the existing literature. This framework guided the systematic retrieval, selection, and synthesis of studies, facilitating a comprehensive understanding of microbial diversity within *Hyalomma* ticks and its implications for tick biology and disease transmission.

### Search strategy

The scientific databases ScienceDirect (https://www.sciencedirect.com/), PubMed (https://pubmed.ncbi.nlm.nih.gov/), Google Scholar (https://scholar.google.com/), and Scopus (https://www.scopus.com) were searched using selected keywords. These keywords were derived from previously published literature. The Boolean operators used to identify relevant studies were as follows: “microbiome” OR “microbial diversity” OR “bacterial community” AND “*Hyalomma*” OR “camel tick” OR “*H. dromedarii*” AND (“MENA” OR “Middle East” OR “North Africa” OR “Algeria” OR “Bahrain” OR “Djibouti” OR “Egypt” OR “Iran” OR “Iraq” OR “Israel” OR “Jordan” OR “Kuwait” OR “Lebanon” OR “Libya” OR “Mauritania” OR “Morocco” OR “Oman” OR “Palestine” OR “Qatar” OR “Saudi Arabia” OR “Somalia” OR “Sudan” OR “Syria” OR “Tunisia” OR “United Arab Emirates” OR “UAE” OR “Yemen”).

### Inclusion criteria

A stringent protocol was followed to determine study inclusion or exclusion. Studies had to fulfill two criteria for inclusion: (1) utilize *16S rRNA* gene sequencing and (2) be conducted within the MENA region. Studies not meeting these criteria were excluded. The initial selection and review were independently performed by M.T.A., followed by a secondary independent revision by M.A.A. Both authors jointly finalized the inclusion decisions. No disagreements arose, eliminating the need for a third reviewer. The inclusion criteria emphasized studies employing 16S rRNA sequencing because it remains the most widely adopted method for microbiome analysis, being both cost-effective and simpler than techniques such as whole-genome sequencing or shotgun metagenomics. In addition, 16S rRNA sequencing facilitates cross-laboratory comparisons due to its reliance on extensively curated bacterial databases, such as Silva. In contrast, whole-genome sequencing typically generates data specific to certain geographical regions, limiting comparability. Furthermore, 16S rRNA data analysis is commonly conducted using free and open-source software like QIIME (Quantitative Insights into Microbial Ecology), developed by the Knight Lab at the University of California, San Diego, USA, which is widely adopted globally by researchers. These advantages establish 16S rRNA sequencing as the preferred method for examining bacterial communities in ticks.

### Exclusion criteria

Studies not meeting the above criteria, i.e., those that did not utilize *16S rRNA* gene sequencing or were conducted outside the MENA region were excluded.

### Data extraction

Data were extracted from the selected studies and entered into a Microsoft Excel spreadsheet (Microsoft Corporation, Redmond, WA, U.S.A.). Columns included details of tick species, host species, study location, study year, identified bacterial genera, and main findings. Data extraction was conducted by M.T.A. and subsequently reviewed by M.A.A. and M.T.A.

### Statistical analysis

Chi-square tests were performed using R software (R Core Team, 2024; version 4.3.3, R Foundation for Statistical Computing, Vienna, Austria) to evaluate associations between the primary bacterial genera and other independent variables, including tick species, host type, and geographical location.

## RESULTS

### Process of article selection

**Figure 1:**
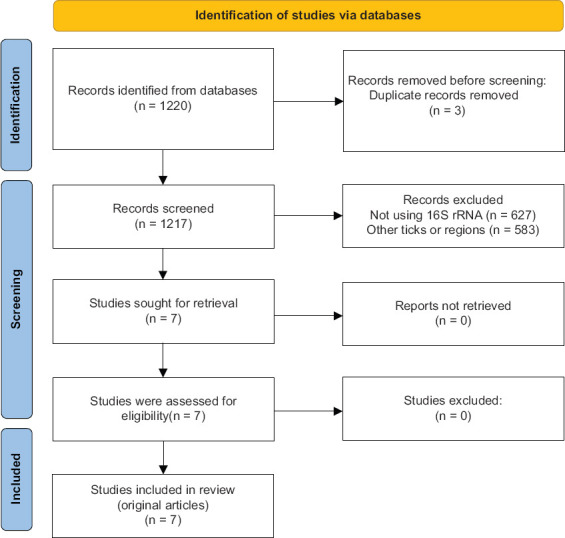
Preferred Reporting Items for Systematic reviews and Meta-Analyseschart showing the methodology for data collection and compilation of scientific research articles using *16S ribosomal RNA* gene sequencing to study tick microbiome in the Middle East and North Africa region from 2014 to 2024.

Initially, 1,220 articles were identified, of which 627 were excluded for not employing *16S rRNA* gene sequencing. An additional 583 articles were excluded as they were outside the MENA region, leaving 10 articles. After eliminating duplicates, seven studies remained, investigating the microbiomes of five *Hyalomma* tick species (Figure 1). These seven studies reviewed involved *H. anatolicum* Koch, 1844, *Hyalomma dromedarii* Koch, 1844, *H. excavatum* Koch, 1844, *Hyalomma scupense* Schulze, 1919, and *H. marginatum* Koch, 1844. All included studies were original research articles, excluding books, book chapters, or review articles.

### Characteristics of the included studies

Among the seven selected studies, four examined *H. dromedarii* (57.14%), two studied *H. anatolicum* (28.57%), and one study covered three tick species: *H. excavatum*, *H. marginatum*, and *H. scupense* (14.29%). Host species included Arabian camels in four studies (57.14%), cattle in two studies (28.57%), and mixed host species (cattle, goats, and sheep) in one study (14.29%). Geographically, three studies were conducted in the UAE (42.86%), two in Saudi Arabia (28.57%), one in Iran (14.29%), and one in Tunisia (14.29%) (Table 1).

**Table 1:** Characteristics of selected studies.

Tick species				Host species sampled			Study site			
		
*Hyalomma dromedarii*	*Hyalomma anatolicum*	Others	Mixed	Camels	Cattle	Mixed	United Arab Emirates	Kingdom of Saudi Arabia	Iran	Tunisia
n = 4 (57.14%)	n = 2 (28.57%)	n = 1 (14.29%)	n = 1 (14.29%)	n = 4 (57.14%)	n = 2 (28.57%)	n = 1 (14.29%)	n = 3 (42.86%)	n = 2 (28.57%)	n = 1 (14.29%)	n = 1 (14.29%)

### Retrieved literature

#### H. anatolicum Koch, 1884

**Figure 2:**
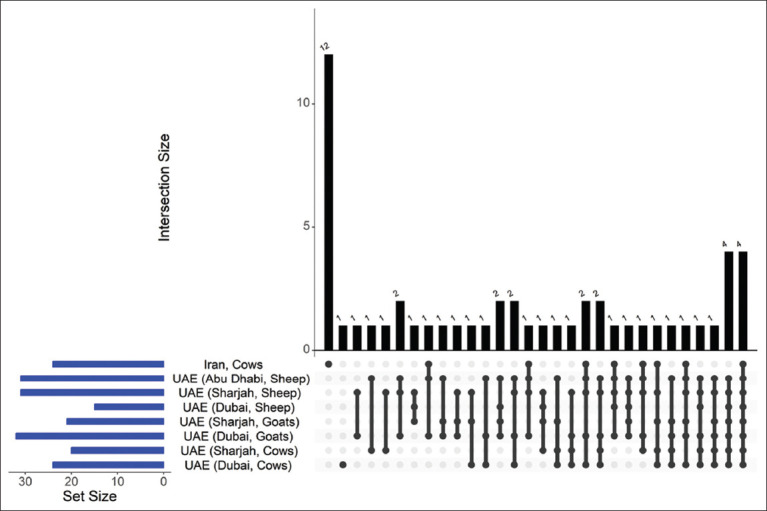
Similarities in the composition of the *Hyalomma anatolicum* microbiome at all locations. The intersection matrix at the bottom indicates shared bacterial genera, with each dot indicating one study. The lines connecting these dots signify similarities. The bars above the matrix show how many genera are common between connected samples, whereas the bars to the left represent the number of genera detected per study. The graph was plotted using R and shows that four bacterial genera were common in all pools and four were common in all pools in the United Arab Emirates.

This tick species is broadly distributed, particularly within but not limited to the MENA region. Under natural conditions, large ungulates such as cattle, camels, goats, horses, and sheep serve as typical hosts [24]. Studies investigating the microbiome of *H. anatolicum* have applied *16S rRNA* gene sequencing in multiple locations, notably the UAE [25] and Iran [26]. Tick specimens were collected from cattle in the UAE (Dubai and Sharjah), goats in Iran and the UAE (Dubai and Sharjah), and sheep in the UAE (Dubai, Sharjah, and Abu Dhabi). The main bacterial genera detected in these studies are presented in Table 2. Despite microbiome variability among these studies, significant overlap in bacterial genera across different geographical locations and host species was evident (Figure 2). Four bacterial genera – *Acinetobacter*, *Corynebacterium*, *Francisella*, and *Staphylococcus* – were consistently found across all studies.

**Table 2:** The most prominent genera in the microbiome of *Hyalomma anatolicum* ticks.

Cow (%)			Sheep (%)			Goat (%)	
		
UAE, Dubai	UAE, Sharjah	Iran	UAE, Dubai	UAE, Sharjah	UAE, Dubai	UAE, Sharjah	UAE, Abu Dhabi
*Proteus* (57.92)	*Staphylococcus* (44.91)	*Francisella* (96.84)	*Cornybacterium* (40.32)	*Francisella* (72.01)	*Staphylococcus* (57.62)	*Acinetobacter* (18.41)	*Corynebacterium* (41.49)
*Bacillus* (14.57)	*Acinetobacter* (22.25)	*Fusobacterium* (0.49)	*Bacillus* (21.12)	Others (9.03)	*Carnimonas* (31.71)	Ignatzschineria (16.23)	*Bacillus* (23.64)
*Enterococcus* (8.16)	*Psychrobacter* (16.53)	*Helococcus* (0.44)	*Streptococcus* (4.07)	*Staphylococcus* (4.12)	*Brevibacterium* (5.41)	*Pseudomonas* (14.47)	*Trueperella* (6.75)

UAE=United Arab Emirates

#### H. dromedarii Koch, 1844

**Figure 3:**
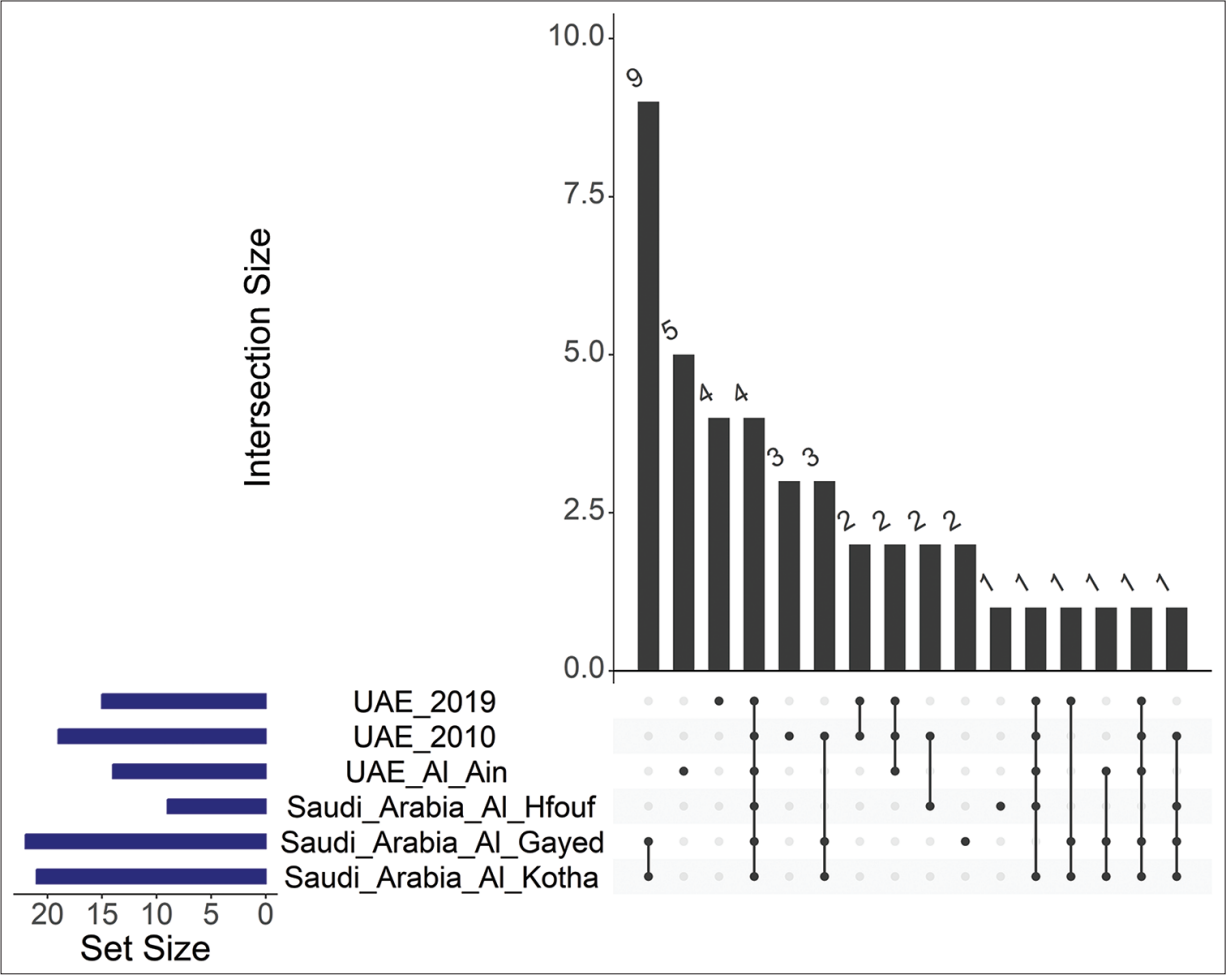
Similarities in the composition of the *Hyalomma dromedarii* microbiome at all locations. The intersection matrix at the bottom indicates shared bacterial genera, with each dot indicating one study. The lines connecting these dots signify similarities. The bars above the matrix show how many genera are common between connected samples, whereas the bars to the left represent the number of genera detected per study. The graph was plotted using R, and it shows that four bacterial genera are common in all studies.

Also commonly known as the camel tick, *H. dromedarii* occurs throughout Africa, the Middle East, and parts of the Far East, particularly in regions where camels serve as its primary hosts; nevertheless, it can also infest other ungulate species [27]. Research examining the microbiome composition of *H. dromedarii* has been conducted across various areas within the UAE and Saudi Arabia. In ticks collected from Al Ain City, UAE, during 2018 and 2019, the bacterial genera *Acinetobacter*, *Corynebacterium*, *Escherichia*, *Francisella*, and *Bacillus* were predominant [9]. In contrast, ticks collected across other regions of the UAE primarily contained microbiomes dominated by *Francisella*, *Staphylococcus*, and *Corynebacterium* [28]. In Saudi Arabia, the composition of microbiomes varied by location: *Pseudomonas*, Marinobacter, and *Proteus* were prevalent in Al Kotha; *Francisella*, *Staphylococcus*, and *Pseudomonas* were dominant in Al Gayed [13]; and *Francisella*, *Proteus*, and *Staphylococcus* predominated in Al Hfouf [29]. Despite these regional variations, there was a notable overlap in bacterial genera among these study sites (Figure 3). Specifically, *Acinetobacter*, *Francisella*, *Pseudomonas*, and *Staphylococcus* were commonly observed across all studied locations. In addition, *Bacillus* appeared in all areas except Al Hfouf, Saudi Arabia, whereas *Corynebacterium* was absent only in Al Gayed, Saudi Arabia.

#### H. excavatum Koch, 1844

*H. excavatum* is primarily found in Africa and certain regions of Asia. Adult stages predominantly parasitize large ungulates, while immature stages (nymphs and larvae) typically infest small mammals [23]. The microbiome composition of *H. excavatum* remains poorly studied; however, research conducted in Tunisia identified several dominant bacterial genera, including *Candidatus Midichloria*, *Pseudomonas*, *Rickettsia*, *Staphylococcus*, *Corynebacterium*, and *Francisella* [30].

#### H. marginatum Koch, 1844

Also known as the Mediterranean *Hyalomma* tick, *H. marginatum* occurs across North Africa, southern Europe, and parts of India. Adult ticks generally parasitize large ungulates, whereas immature ticks typically infest small mammals [27]. Microbiome studies for this tick within the MENA region are limited. Nonetheless, a study from Tunisia reported a microbiome predominantly composed of *Candidatus Midichloria*, *Pseudomonas*, *Rickettsia*, *Staphylococcus*, *Corynebacterium*, and *Francisella* [30].

#### H. scupense Schulze, 1919

*H. scupense* is a two-host tick mainly distributed throughout the Middle East, Africa, and southern Europe [31], primarily infesting artiodactyl hosts [32]. Investigations into the microbiome composition of *H. scupense* using 16S rRNA sequencing within the MENA region are sparse. However, one study indicated variation within the microbiome, with *Francisella* and *Rickettsia* as predominant genera across adult ticks (both males and females), nymphs, and eggs [30] (Table 3).

**Table 3:** Composition of the microbiome of *Hyalomma scupense* in eggs, nymphs, and adults of both genders using *16S ribosomal RNA* gene sequencing.

Females	Males	Nymph	Eggs
*Ricktessia*	*Escherchia- Shigello*	*Rickettsia* genre	*Ricktessia*
		*Incertae sedis*	
*Francisella*	*Ricktessia*	*Ricktessia*	*Francisella*
*Rickettsia* genre	*Francisella*	*Francisella*	*Midichloria mitochondrii*
*Incertae sedis*			

### Identified literature gaps

Published studies have been identified in only four of the 23 countries within the MENA region. All these studies addressed the microbiome of *Hyalomma* ticks on a localized scale rather than providing national coverage. In addition, microbiome variations in relation to tick and host species, tick lifecycle stages, and temporal and spatial factors were explored only for *H. anatolicum* in the UAE, *H. scupense* in Tunisia, and *H. dromedarii* in the UAE and Saudi Arabia, respectively. Consequently, even in countries where studies have been conducted, there remains limited data regarding the impact of biotic and abiotic factors on the microbiome composition of ticks (Table 4).

**Table 4:** Gaps identified in the literature.

Country	Sampling level	Microbiome composition	Differences between tick species	Host-driven variations	Lifecycle– driven variations	Spatial variations	Temporal variation	Tick gender– driven variations	Reference
Saudi Arabia	Local	+	-	-	-	+	-	-	[13]
UAE	Local	+	-	+	-	-	+	-	[24]
Iran	Local	+	-	-	-	-	-	-	[25]
Tunisia	Local	+	+	-	+	-	-	+	[29]
Other MENA Countries	N/A	-	-	-	-	-	-	-	N/A

N/A=Not Applicable, +=Investigated, -=Not investigated, UAE=United Arab Emirates, MENA=Middle East and North Africa

## DISCUSSION

In many regions where *Hyalomma* ticks are prevalent, their microbiome remains insufficiently investigated, highlighting the urgent need for further research on these important vectors. This review assessed available microbiome studies on five *Hyalomma* tick species using *16S rRNA* gene sequencing, identifying eight bacterial genera commonly observed either across different tick species or within the same species across multiple studies.

### Acinetobacter

Although *Acinetobacter* is primarily recognized as an environmental bacterium frequently found in soil [33, 34], its detection in host blood and various tick tissues suggests active circulation between ticks and their hosts [35]. This hypothesis is supported by the pathogenic [36–38] and antibiotic-resistant nature of this genus [37, 38]. *Acinetobacter* was consistently identified in all studies involving *H. dromedarii* and *H. anatolicum*, raising potential concerns about its pathogenicity, particularly in regions with dense host populations.

### Bacillus

*Bacillus* was observed in *H. dromedarii* ticks across most studied locations, with the exception of Al Hfouf, Saudi Arabia. This genus may play a significant role in camel ticks, as certain *Bacillus* species are known to reduce the pathogenicity of *Borrelia* in other tick species [10]. This effect may explain the absence of *Borrelia* in the reviewed studies. Moreover, *Bacillus* genus have previously been identified within *Rhipicephalus microplus* ticks [39].

### Flavobacterium

*Flavobacterium* has repeatedly been detected across various tick species, including *H. dromedarii* [40–44]; however, its precise role within tick microbiomes remains unclear, as it is rarely discussed explicitly in the literature.

### Francisella

*Francisella* was documented in all reviewed *Hyalomma* species, indicating its critical role in tick survival. *Francisella*-like endosymbionts are vertically transmitted [45] and constitute major components of the *Hyalomma* microbiome globally. They have been recognized as primary endosymbionts across diverse *Hyalomma* species and hosts in Europe, Asia, and Africa [8]. *Francisella* likely contributes to tick homeostasis [9] and Vitamin B synthesis [8]. Notably, pathogenic *Francisella* species, including *Francisella tularensis*, have been responsible for disease outbreaks in Europe and America and are recognized as tick-borne pathogens [46, 47]. Although *F. tularensis* was not specifically identified in some studies on *Hyalomma* ticks, it has been documented in other hard tick species [46].

### Rickettsia

*Rickettsia* was found in *H. marginatum* and *H. excavatum* ticks, as well as in eggs, nymphs, and adult males and females of *H. scupense*. These findings underscore the potential nutritional significance of Rickettsial endosymbionts in these tick species [11]. Pathogenic *Rickettsia* species known to be transmitted by *Hyalomma* ticks include *Rickettsia* *conorii* in *H. dromedarii* [47], *Rickettsia aeschlimannii* in *H. marginatum*, and *Rickettsia massiliae* in *Hyalomma aegyptium* [46]. In addition, *Rickettsia* frequently co-occurs with *Francisella*-like endosymbionts within the *Hyalomma* microbiome [8].

### Pseudomonas

*Pseudomonas* was consistently present in all sampled *H. dromedarii* ticks and might possess immunological significance, potentially reducing colonization by pathogens such as *Borrelia burgdorferi*. This genus could be acquired and maintained throughout the tick’s lifecycle [34].

### Staphylococcus and Corynebacterium

*Corynebacterium* and *Staphylococcus* were detected across all studies involving *H. anatolicum* and *H. dromedarii*, respectively. These bacteria are likely acquired from environmental sources and retained during the tick’s lifecycle [34]. Although their precise roles remain uncertain, pathogenic species of *Staphylococcus*, such as *Staphylococcus lentus* and *Staphylococcus saprophyticus*, previously identified in *Hyalomma* ticks [48], may contribute to their frequent detection.

### Hyalomma ticks and their microbiome

The microbiome of ticks encompasses various commensal, pathogenic, and symbiotic bacterial species, significantly influencing tick fitness, immunity, physiology, and vector competence [49]. *Hyalomma* ticks are established vectors of critical tick-borne diseases, including CCHF, *Anaplasma*, and *Theileria*, causing significant conditions such as anaplasmosis and pyroplasmosis [50]. Historical outbreaks of *Hyalomma*-associated CCHF in the MENA region and recent increases in CCHF cases emphasize the public health significance of these ticks [8]. Understanding pathogen-symbiont interactions within *Hyalomma* tick microbiomes is essential for developing effective disease management strategies. The broad host range of *Hyalomma* ticks, which includes sheep, cattle, camels, goats, donkeys, and horses, alongside their role in disease transmission [51], underscores the economic and veterinary importance of these ticks. *Hyalomma* infestations negatively impact livestock productivity and health, reinforcing the need for comprehensive microbiome research and targeted tick-control measures [52]. Targeting prominent bacterial genera, especially *Francisella*, using microbiome-specific vaccines and targeted antibiotics [49] could provide effective management solutions against *Hyalomma* ticks in the MENA region.

### Recommendations

Given the widespread distribution of *Hyalomma* ticks and their associated animal hosts across the MENA region, the existing gap in microbiome research represents a significant missed opportunity for impro-ving public and veterinary health. Because microbiome composition can fluctuate due to environmental and host-related factors, conducting periodic and longitudinal studies is crucial to monitor these shifts and to associate them with disease outbreaks or altered tick-host interactions. Investigating microbiome diversity throughout the various MENA countries may offer critical insights into the regional epidemiological effects on animal health, as cross-country studies could uncover unique microbial communities adapted to specific local environments, thus influencing pathogen transmission dynamics. In addition, implementing long-term studies that capture seasonal and inter-annual microbiome variations could substantially enhance our understanding of how climate and human activities affect disease transmission patterns. Establishing a regional database and encouraging collaborative efforts among MENA countries would enable the creation of a comprehensive data repository, fostering deeper insights into microbiome patterns. Further research into host-specific and environmental determinants, such as vegetation types, climate conditions, and land-use patterns, could help identify key drivers influencing microbiome diversity and pathogen presence in *Hyalomma* ticks. In addition, examining interactions between pathogens such as *Rickettsia* and other microbiome constituents could clarify how the composition of the tick microbiome influences pathogen transmission, highlighting microbial species that either inhibit or promote pathogen survival and transmission. Furthermore, studying the microbiome across different ecosystems and habitat gradients is essential for understanding variations within *Hyalomma* tick populations across their geographical range. Finally, developing an online database encompassing microbiome studies of different *Hyalomma* species, their potential host animals, import and export routes of these host species, and associated tick-borne diseases would significantly enhance research accessibility and facilitate informed decision-making.

## CONCLUSION

This systematic review provided a comprehensive analysis of existing microbiome research on *Hyalomma* ticks within the MENA region, utilizing next-generation sequencing of the *16S rRNA* gene. Key findings indicate the consistent presence of eight bacterial genera – *Acinetobacter*, *Bacillus*, *Flavobacterium*, *Francisella*, *Rickettsia*, *Staphylococcus*, *Pseudomonas*, and *Corynebacterium* – across multiple tick species and geographic locations. These bacteria potentially play significant roles in tick physiology, pathogen transmission dynamics, and overall tick ecology. Notably, *Francisella* emerged as a predominant genus, suggesting its importance for tick survival and homeostasis, whereas the consistent presence of potentially pathogenic genera such as *Acinetobacter* raises concerns for animal and public health.

The strengths of this review include its systematic approach, stringent inclusion criteria, and critical evaluation of microbiome data across multiple tick species, hosts, and locations, providing a foundational reference for future research. Nonetheless, several limitations should be acknowledged: the scarcity of published data from the majority of MENA countries, the restriction of analysis to genus-level identification due to the methodological limitations of 16S rRNA sequencing, and the inability to explore microbiome variability comprehensively across temporal and spatial gradients.

Future research should address these identified gaps through broader geographic coverage, detailed investigation of host-microbiome interactions, and longitudinal studies to examine the influence of environmental changes and human activity on microbiome composition. Integrating advanced genomic methodologies, such as whole-genome sequencing and metagenomics, could further refine microbial identification and functional characterization. In addition, establishing regional collaborations and databases for continuous data sharing could significantly enhance understanding of tick microbiomes and facilitate targeted management strategies. Ultimately, bridging these knowledge gaps will inform effective public health interventions and advance veterinary care practices, reducing the substantial health and economic burdens imposed by tick-borne diseases in the MENA region.

## AUTHORS’ CONTRIBUTIONS

MAA: Conceptualization and project administration. MTA and MAA: Methodology, writing – original draft preparation, and writing – review and editing. MTA: Investigated and visualized the study. All authors have read and agreed to the published version of the manuscript.
